# Supplementary material for: Identification of photoperiod-induced specific miRNAs in the adrenal glands of Sunite sheep (Ovis aries)
Source: Front Vet Sci. 2022 Jul 22;9:888207. doi: 10.3389/fvets.2022.888207 (PMC9354845; doi:10.3389/fvets.2022.888207)
Supplement: Supplementary file 1 [file Data_Sheet_1.ZIP › Supplementary file/miRDeep2 file.zip!/miRDeep2 file/Know_Structure/LP42A1/oar-mir-3958.pdf]

miRBase precursor : oar-mir-3958
Total read count : 11851
oar-miR-3958-5p read count : 17
oar-miR-3958-3p read count : 11834
remaining reads : 0

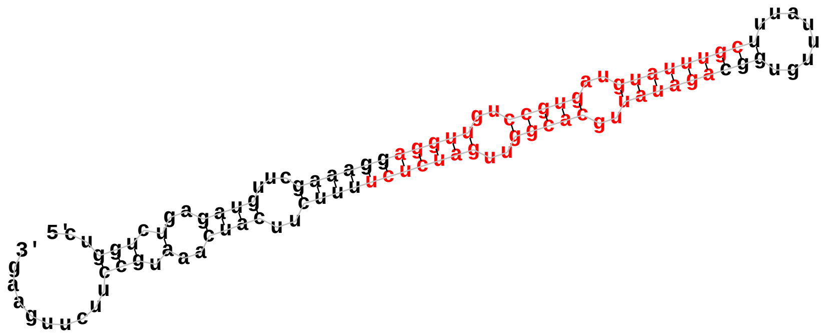

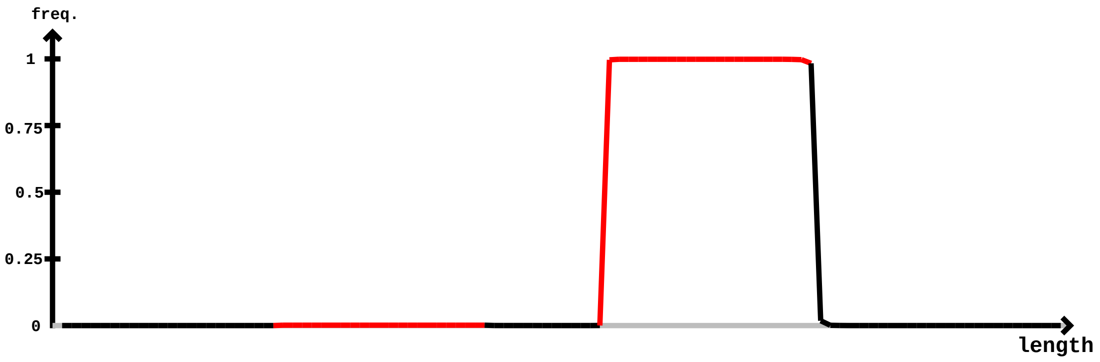


```
                                                      oar-miR-3958-3p
                          oar-miR-3958-5p
5'- cuggucugagauguucgaaaggagguuguccgugauguauuugcuuuauuuguggcagauauugcacgguugaucucuuuucuucaucaaaugccuucuugaag   -3'    exp
    ..(((.(..((((...(((((((((((..(((((..(((((((((........)))))))))..)))))..)))))))))))..))))..).))).........    reads     mm        sample
    ......................agguuguccgugauguauuugc............................................................    15        0         seq
    ......................agguuguccgugauguauuugcu...........................................................    1         0         seq
    ......................agguuguccgugauguauuugcuuuau.......................................................    1         0         seq
    .......................................................cagauauugcacgguugaucucu..........................    4         0         seq
    ........................................................agauauugcacgguugauc.............................    6         0         seq
    ........................................................agauauugcacgguugaucu............................    13        0         seq
    ........................................................agauauugcacgguugaucuc...........................    161       0         seq
    ........................................................agauauugcacgguugaucucu..........................    11409     0         seq
    ........................................................agauauugcacgguugaucucuu.........................    203       0         seq
    ........................................................agauauugcacgguugaucucuuu........................    9         0         seq
    ........................................................agauauugcacgguugaucucuuuu.......................    3         0         seq
    .........................................................gauauugcacgguugaucuc...........................    1         0         seq
    .........................................................gauauugcacgguugaucucu..........................    24        0         seq
    ............................................................auugcacgguugaucucu..........................    1         0         seq
```
